# Fs-Ablated Trenches on the Surface of Microsphere for Whispering Gallery Modes Cleaning

**DOI:** 10.3390/mi17030381

**Published:** 2026-03-21

**Authors:** Hiba A. Rizk, Viktor A. Simonov, Vadim S. Terentyev, Vladislav E. Fedyaj, Andrey E. Simanchuk, Alexander V. Dostovalov, Sergey A. Babin

**Affiliations:** Institute of Automation and Electrometry, 1 Ac. Koptyug Ave., Novosibirsk 630090, Russia; rizkh@iae.nsk.su (H.A.R.); terentyev@iae.nsk.su (V.S.T.); fedyajve@iae.nsk.su (V.E.F.); simanchuk@iae.nsk.su (A.E.S.); dostovalov@iae.nsk.su (A.V.D.); babin@iae.nsk.su (S.A.B.)

**Keywords:** spherical microresonator, whispering gallery modes, spectral cleaning, femtosecond laser ablation

## Abstract

This study addresses the problem of whispering gallery mode (WGM) selection in spherical microresonators by means of their femtosecond micro-processing. The proposed method involves fabrication on the microsphere surface of defects playing the role of scattering elements for higher-order modes with low azimuthal mode indices. These two T-shaped trenches are created using femtosecond laser ablation, with a depth of 2 microns, gap of 30 microns between them, and each of length of 20 microns along the equatorial direction. A tapered fiber with a sub-micron waist diameter serves as the excitation element for WGMs. This method allows for spectral purification of the WGMs, reducing the number of resonances by 180 times, with a quality factor of $Q>10^{5}$ for the non-inverted spectrum in the form of resonance dips. Additionally, an inverted spectrum with narrow resonance peaks of about 35%, low background level and single mode regime with 3 dB side peak suppression has been simultaneously achieved in the taper transmission, for the first time to our knowledge. The latter was obtained by exciting the microsphere at the taper waist. These results hold promise for the development of narrowband filters, laser mode selectors, and optical sensors based on microresonators.

## 1. Introduction

Axially symmetric and spherical microresonators in particular, are widely used in various fields of optics, such as narrowband filters [1], lasers [2,3], optical sensors [4], and nonlinear optics [5,6]. Spherical microresonators have an advantage over bottle or SNAP resonators due to their smaller mode volume, which is preferable for nonlinear optics applications, as it reduces the thresholds for nonlinear effects.

The process of manufacturing microspheres is comparatively less labor-intensive than that of other types of resonators with a small mode volume, such as disk microresonators [7]. By melting the tip of an optical quartz fiber using a laser or plasma fiber splicer, one can fabricate resonator for filtering radiation with very high optical characteristics. These include low losses, which ensure high quality factor ($Q>10^{6}$), and the ability to achieve a specified radius with high repeatability.

Excitation of a spherical microresonator’s WGMs is most conveniently achieved using a tapered fiber. In this context, the taper–microsphere coupling system offers wide capabilities. Thanks to this system it is possible to obtain, firstly, a standard “non-inverted” interference pattern in the taper transmission, appearing as intensity minima (dips). Secondly, one can observe back-reflection generated by weak internal scattering within the microsphere, as well as backscattering in the contact region between the taper and the microsphere, the peak maximum of which, in the case of a high Q-factor ($Q>10^{8}$), can reach tens of percent [8]. Thirdly, one can obtain a non-standard interference pattern in transmission in the form of asymmetric profile (Fano resonances) [9]. Among the possible forms of Fano resonances, one can distinguish the “inverted” interference pattern, which is manifested as narrow peaks on a low background [10,11].

One of the disadvantages of spherical microresonators is the presence of a very large number of excited modes, which arise from the optical conversion of taper modes into modes of the microsphere. The problem of mode cleaning in this optical system is quite critical and is currently being actively studied [12,13,14]. Mode selection can be performed in two spatial directions, radial [15] and polar [16], using thin metal absorbing film, as was demonstrated in our previous works theoretically and experimentally [11].

A simple method of creating micro-scatterers on the surface of a microsphere is described in work [17], aimed at introducing high losses for modes with lower azimuthal indices. The primary goal of our present study is to expand the capabilities of this method for mode cleaning and for obtaining various types of response functions (resonance lineshapes profiles) in the transmission spectrum of the taper–microsphere coupling system. In particular, it is of interest to obtain, in addition to the typical non-inverted pure spectrum, an inverted spectrum with resonant peaks for the first time to our knowledge. This would be useful for applications in mode selection of lasers with ring resonators, as well as for the development of optical sensors and narrowband spectral filters.

## 2. Basic Principles

**Figure 1:**
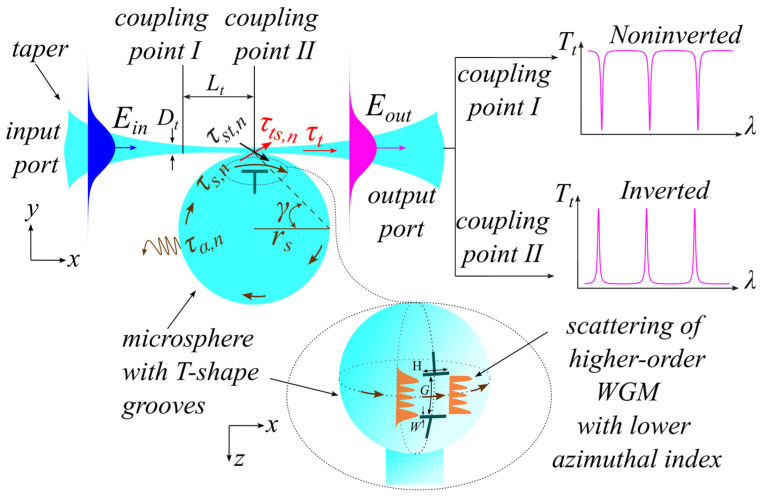
Scheme of taper-spherical microresonator coupling system.

The studied problem is schematically presented in Figure 1.

The main idea of this method is based on the scattering of higher-order modes, i.e., the occurrence of high losses for modes with lower azimuthal indices, which have wider field distribution in the polar direction along the microsphere surface (inset on Figure 1). This is achieved by applying scatterers in the form of trenches on the surface, the spacing between which is carefully controlled to avoid scattering losses for the fundamental mode and all modes with maximum azimuthal index. This reduces the Q-factor of unwanted modes, thus effectively suppressing them.

On one hand, the degree of insertion losses into the resonator for higher-order modes, whose intensity peaks increase in both the polar and radial directions, depends on the trench depth. The losses grow with increasing trench depth, thus enhancing the mode cleaning. On the other hand, this increases probability of introducing losses for fundamental mode of the microsphere due to the internal stress formation in the vicinity of the impact site, since the trenches are created by ablation of the microsphere surface with a femtosecond laser.

The most commonly used excitation element is tapered fiber due to its efficiency and native light input and output in experimental setups. To achieve optimal taper transmission, it is crucial that the taper has an adiabatic profile [18], i.e., the transformation of the optical fiber modes into the fundamental mode of the taper’s narrow section must occur without the excitation of higher-order modes. This is expressed in a flat taper transmission spectrum (transmission variation and average losses < 10% in the range of 1540–1560 nm). It is also significant whether the taper touches the microsphere or is positioned at a small distance, as this affects the coupling coefficients between the taper and microsphere modes, the resulting Q-factor, and the contrast of the spectral resonances. In this work, the taper is put in physical contact with the microsphere, and on the one hand, this leads to increased losses in the resonator, but on the other hand, the entire system becomes more stable with respect to external influences.

## 3. Phenomenological Theory

It is possible to characterize the optical properties of the whispering gallery modes supported by microspheres using coupled-mode theory [19] through their excitation by efficient coupling to an evanescent near-field wave. This is achieved through maximum spatial overlap of this wave field with the whispering gallery mode fields and perfect phase matching between them. To explain the taper–microsphere optical system, it can be considered practically equivalent to a two-mirror multibeam reflection interferometer, where the coupling strength between the fundamental taper mode and the whispering gallery modes plays a role similar to the transmittance coefficients of the interferometer mirrors.

Figure 1 shows the taper–microsphere coupling system, in which an optical wave with a field $E_{in}$ from the input port propagates along the taper. Part of this wave passes through it with an amplitude coefficient of the transmitted field $\tau_{t}$ without coupling to the microsphere, thus forming the spectrum background, while the other part excites N whispering gallery modes in the microsphere by coupling to them with the amplitude coefficient $\tau_{st,n\;}$ for each n-th mode at the coupling region/point. The excited mode circulates in the microsphere with an amplitude transmission coefficient $\tau_{s}$ via total internal reflection and returns to the coupling region/point after the round-trip. Part of this field couples to the taper with the amplitude coefficient $\tau_{ts,n\;}$ and combines with the zero beam passing through the taper to output port, thus forming output field $E_{out}$, while the remaining portion propagates further within the microsphere, then partially returns again by the same optical coupling mechanism to the taper, and so on. As a result, the optical fields of each mode within the microsphere are summed up in the taper after each round-trip. The optical interaction that occurs in the coupling region between the microsphere and the taper can be represented as follows:

(1) $${\tilde{\tau }}_{t}=\frac{E_{out}}{E_{in}}=\tau_{t}+\sum_{n=1}^{N}\frac{\;\tau_{st,n\;}\tau_{ts,n\;}\tau_{\alpha ,n\;}\;{\;e}^{-i\psi_{n}}}{1-\tau_{s,n\;}\;\tau_{\alpha ,n\;}\;{\;e}^{-i\psi_{n}}}$$

Note that formula (1) implies only one mode in the taper, but if there is more than one, it is necessary to add each mode’s own sum for each mode of the taper according to the modes of the microsphere. In addition to the transmission of the taper ${\tilde{\tau }}_{t}$ in the taper–microsphere system, there is also a small amount of reflection and backscattering, which is not given here, although it is used, for example, to narrow and to stabilize the spectrum of laser radiation [20]. For simplicity, we will consider that optical coupling occurs between the taper mode with only one WGM, and thus the spectral transformation at the taper output is determined from (1) by the response function of the taper–microsphere system as follows:

(2) $$\tilde{T_{t}}={\left|{\tilde{\tau }}_{t}\right|}^{2}=T_{t}+\frac{2}{T_{st}}\sqrt{\frac{T_{t}}{T_{\alpha }}}\left(\cos\left(\varphi +\vartheta \right)-\sqrt{T_{s}T_{\alpha }}\cos\left(\vartheta \right)\right)A(\varphi )+A(\varphi )\;A(\varphi )\;=\;\frac{{T_{st}}^{2}\;T_{\alpha }}{1+T_{s}T_{\alpha }-2\sqrt{T_{s}T_{\alpha }}\cos\left(\varphi \right)}$$

Here the intensity coefficients of the taper, microsphere and coupling coefficient are related to the amplitude coefficient as $\tau_{t,s}=\sqrt{T_{t,s}}\;e^{i\Phi_{t,s}}$ and $\tau_{ts}=\tau_{st}=\sqrt{T_{st}}\;e^{i\Phi_{st}}$. The transmission coefficient of the resonator for one round-trip is $T_{\alpha }=\tau_{\alpha }^{2}=e^{-\alpha L_{s}}$, $\Phi_{t,s}$ and $\Phi_{st}$ are the phases of the amplitude coefficients, $\varphi =\frac{{2\pi nL}_{s}}{\lambda }-\Phi_{s}$ is the phase for the round-trip and $\vartheta =\Phi_{t}+\Phi_{s}-2\Phi_{st}$ is the total phase (Hamy phase), α is the coefficient of linear losses in the microsphere and $L_{s}=2\pi r_{s}n_{s}$ is the optical length of the resonator and $n_{s}$ is the effective refraction index of the microsphere’s mode.

The first term of Equation (2) defines the background transmission level, the value of which varies depending on the coupling point with the microsphere along the taper. The second term indicates the asymmetry (resulting from the inclusion of the Hamy phase), while the third term indicates the symmetric response function of the Airy type. The magnitude and sign of the resonance asymmetry are determined by $T_{t}$ and ϑ, respectively.

It follows from formula (2) that to obtain an asymmetric Fano resonance, only one microsphere mode is sufficient [10], instead of two, which are required in an analytical form based on shortened equations [9].

**Figure 2:**
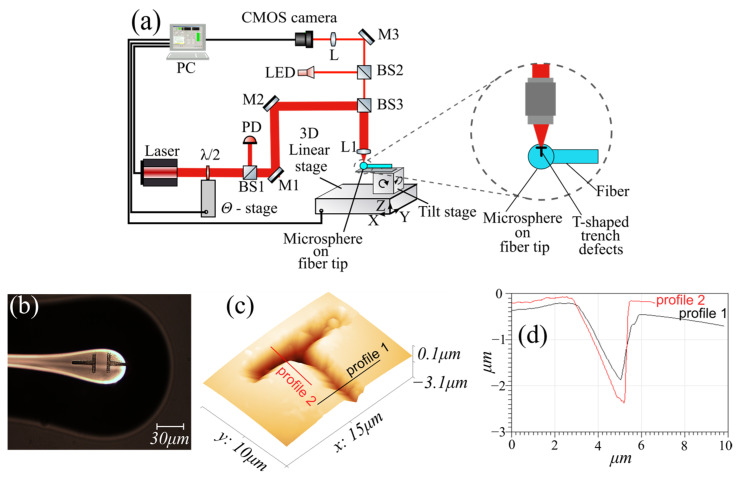
(**a**) FS laser modification setup, (**b**) microsphere with trenches, (**c**) AFM-profile of trenches and (**d**) Profiles dimensions of the trench.

From the taper–microsphere system’s response function it is possible to determine the characteristics and the shape of the taper output’s transmission spectrum based on certain specific cases (Figure 2). For the first one, the microsphere contacts the taper in the coupling point I, which is distant from its waist, and where the taper diameter is sufficient to prevent significant scattering losses. The background intensity $T_{t}$ will approach unity. In this case, the condition of total internal reflection (TIR) is satisfied at a constant angle of incidence $\gamma >\gamma_{TIR}$, resulting in a symmetric transmission spectrum in the form of resonant dips (non-inverted spectrum) with phase $\vartheta =\left(2m+1\right)\pi ;\;m\;=\;0,\;1,\;2,\;\ldots$, which represents a typical shape for the transmission spectrum of the response function of this system.

For the second case, contact occurs at coupling point II exactly at the taper’s center, and the diameter of the taper is small enough to scatter light in all directions, but mostly inside the microsphere. In this case, the value of $T_{t}$ approaches zero, and the primary portion of light strikes the microsphere surface at an angle contrary to the TIR condition $\gamma <\gamma_{TIR}$ and exits the microsphere ($\tau_{\alpha ,n}\to 0$), while the remaining portion continues its path within it (for more information, see the Figure S1a,b in Supplementary Materials). As a result, in the case of one microsphere mode, only the last term of Equation (2) remains, and a transmission spectrum is formed as resonance peaks (inverted spectrum) with an asymmetric profile if the phase $\vartheta \neq \left(2m+1\right)\pi$. The latter becomes possible when there is significant scattering in the region where the taper is connected to the microsphere, which leads to a change in at least the phase of $\Phi_{t}$. The loaded quality factor of the microresonator according to Equation (2) is:

$$Q=\frac{\lambda }{FSR}F\approx \frac{L_{s}}{\lambda }\frac{\pi {\left(T_{s}T_{\alpha }\right)}^{1/4}}{1-\sqrt{T_{s}T_{\alpha }}}$$

where F is the finesse and the internal ($Q_{\alpha }$) quality factors can be obtained by setting $T_{s}=1$.

## 4. Experimental Setup

The process of microsphere manufacturing was carried out by cutting SMF-28e fiber and fusing its tip by an electrical discharge of FSM-100P (Fujikura, Tokyo, Japan) arc fusion splicer, to form the microsphere with diameter of 160 μm due to surface tension (Figure 2b). The ratio of the microsphere axes was 0.95 (eccentricity e = 0.31).

Next, the microsphere was placed on a femtosecond laser modification setup (Figure 2a), which uses the laser ablation process to create microchannels. For this purpose, radiation from a Pharos 6W FS laser (Light Conversion, Vilnius, Lithuania) with a wavelength of 1.026 µm, a pulse duration of 230 fs, a pulse repetition frequency of 200 kHz, and a pulse energy of 1 µJ was focused using a 50× PLAN APO NIR HR (Mitutoyo, Kawasaki, Japan) (NA = 0.65) microscopic lens on the surface of a microsphere, which was moved using a high-precision ABL1000 (Aerotech, Pittsburgh, PA, USA) 3-axis positioner. The positioner followed a predetermined trajectory at a speed of 5 μm/s, so as to form a T-shaped defect in the form of trenches, the depth of which depended on the pulse energy. To increase the depth of the trenches, the modification was carried out in several passes along the same trajectory. The modification was positioned relative to the center of the microsphere using an optical imaging system using the same microscopic lens that was used for ablation. The dimensions of the modifications (Figure 2b) in the shape of two letters “T”, optimal for cleaning the modes [17], were chosen as follows: W = 3 μm, H = 20 μm, and the varied gap between them, G = 20,25,30 μm. The latter was chosen to be larger than the size of the microsphere’s main mode in the polar direction. The depth of the trenches was about 2 microns as shown by atomic force microscope (AFM) MultiView 2000 (Nanonics Imaging, Jerusalem, Israel) (Figure 2c,d).

**Figure 3:**
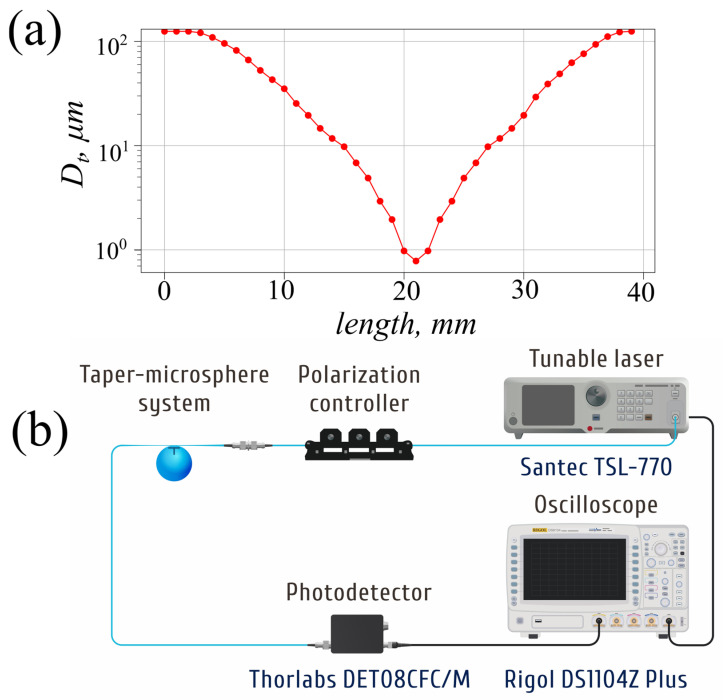
(**a**) Profile of taper diameter. (**b**) Scheme of measurement setup: blue color is for optical fiber, black is for electric wires.

The taper is the key element for changing the shape of Fano resonances. It was manufactured at the Lightel CW 200B (Lightel Technologies, Renton, WC, USA) fiber coupler machine. Fabricated tapers demonstrate low losses (<0.5 dB), uniform (<10%) transmission spectrum over a wide spectral range (50 nm) and high repeatability of properties. The characteristic dependence of the taper diameter along its length is shown in Figure 3a. The microsphere was oriented so that the contact point with the taper was located in the middle between the trenches.

The experimental setup used to measure transmission spectra (Figure 3b) consists of a scanning laser Santec TSL-770 (Minamikomagun, Japan) as a light source with a linewidth of 60 kHz. The light passes a manual fiber polarization controller equipped with rotating paddles, allowing for separate excitation of TE and TM WGMs. After exiting the taper–microsphere system, the polarized light is detected with photo-detector Thorlabs DET08CFC/M to be recorded with oscilloscope Rigol DS1104Z Plus (Shanghai, China). The laser generates syncro-pulses with certain wavelength steps during sweeping, allowing for the reconstruction of the wavelength from the oscilloscope trace.

## 5. Experimental Results

**Figure 4:**
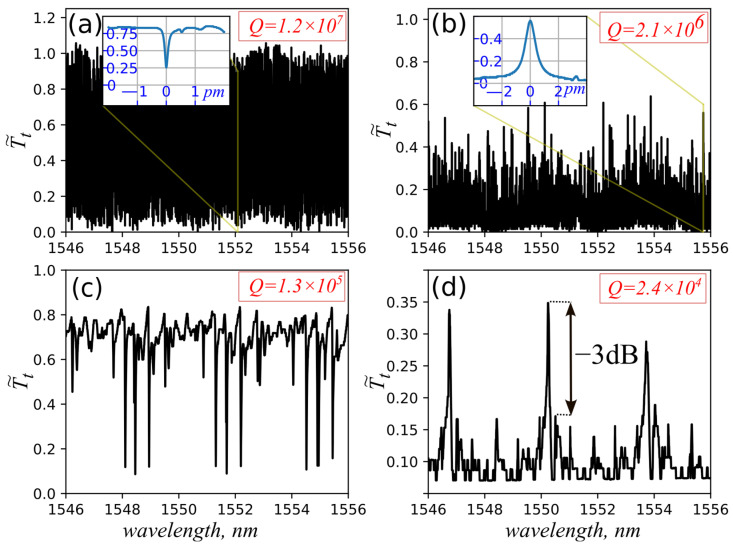
Transmission spectra $\tilde{T_{t}}$ for an unmodified microsphere: (**a**)—$L_{t}$ = 1.5 mm, (**b**)—$L_{t}$ = 0 mm. For modified microsphere: (**c**)—$L_{t}$ = 1.5 mm, (**d**)—$L_{t}$ = 0 mm.

Figure 4 shows the measured typical transmission spectra of an unmodified microsphere (Figure 4a,b). The resulting spectra for the modified microsphere are shown in Figure 4c,d. They were measured at two coupling points: I—away from the taper’s center resulting in non-inverted spectrum (Figure 4a,c) and II—at the taper’s center for inverted spectrum (Figure 4b,d), respectively.

The taper touching point may not be exactly the same before and after modification, as the microsphere may have been rotated around its axis during the process. In the direction transverse to the mode plane, the taper touching point of the unmodified microsphere aligned with an accuracy of about 10 microns relative to the center between the grooves, which did not significantly affect the spectral parameters.

The transmission spectra of unmodified microsphere show dense resonances resulting from the excitation of a large number of optical modes. This is due to the large size of the microsphere, which can support them, in addition to the excitation of non-degenerate modes due to the sphere’s deviation from a spherical shape. The number of modes that meet the rule $\left|T_{t}-{\tilde{T}}_{t,max(min)}\right|>0.1$ was counted, and based on this, the number of excited modes reached 1300 for the non-inverted spectra with maximum Q-factor = $1.2\times 10^{7}$ within a free spectral range of 3.3 nm (Figure 4a). The inverted spectra show a reduction in the mode’s number to 316 due to significant losses for higher-order modes because of the small angle of incidence on the inner surface of the microsphere $\gamma <\gamma_{TIR}$ (Figure 4b).

The results for the microsphere with trenches illustrate a spectral cleaning effect with a pure separation of resonant modes by free spectral range that corresponds to scattering of the higher-order WGMs with lower azimuthal indices while passing through the trenches. The intensity of their peaks increases in the polar direction along the microsphere surface parallel to the stem of “T”-shaped trenches, thus effectively suppressing them (Figure 1). Conversely, the controlled distance between the trenches maintains the excitation of fundamental mode and some modes propagate near the equator with sizes less than the gap width. Consequently, the number of excited modes was reduced by 180 times for the non-inverted spectrum and by 60 times for the inverted spectrum, with quality factors reduced to $Q=1.3\;\times \;10^{5}$ and $Q=2.4\times 10^{4}$, respectively. The decrease in the number of resonances is approximately the same as in [11], which used thin absorbing film. Since the maximum Q-factors correspond to the modes with the maximum contrast in Figure 4c, it can be argued that they represent a group of modes with maximum azimuthal but different radial indices, including the fundamental mode, as they experience minimal loss. According to the calculations of the overlap integrals (see the Supplementary Materials, Figure S2), all other modes with lower azimuthal indices should have smaller Q-factors. For the inverted spectrum in Figure 4d, maximum $Q=9.4\times 10^{4}$ is for one of the side peaks. However, the losses introduced into the microsphere in the case of the taper waist seem to increase the losses in the resonator. This leads to a more efficient excitation of a mode with greater losses and $Q=2.4\times 10^{4}$. As a result, a mode with a lower azimuthal index may have a higher transmission coefficient.

Microspheres with different gap size G were manufactured, but the best results in terms of spectral cleaning parameters (high Q-factor and mode suppression) were obtained for G = 30 μm, as shown in Figure 4. In particular, single mode regime and suppression of side peaks by 3 dB were obtained for the inverted spectrum (Figure 4d). The following estimated parameters were obtained for the remaining microspheres (Table 1):

## 6. Discussion

As follows from Figure 4, the scattering trenches are quite effective for cleaning microsphere modes. At the same time, the depth of the trenches is not so deep compared to the results of [17]: about 2 μm instead of 8 μm, as follows from Figure 2d.

In addition to the standard non-inverted spectrum, an inverted spectrum can be obtained. The shape of the peaks in transmission with a maximum transmission coefficient of tens of percent is by orders of magnitude higher than the maximum peak of reflection from the taper–microsphere system with relatively low quality factors ($Q<10^{7}$), so it can be used more efficiently to create feedback in various schemes of laser resonators. In addition to the main two forms of the spectra shown in Figure 4, intermediate (“transitional”) forms of Fano resonances can be obtained by varying the point of contact between the taper and the microsphere ${0\;mm<L}_{t}<1.5\;mm$.

It should be noted that an “imperfect” microsphere is used in this work, that has a noticeable ellipticity, which leads to the appearance of additional resonances due to non-degenerate modes with lower azimuthal index. Nevertheless, even under these conditions, this method greatly reduces the number of resonances.

Among the many possible selection methods, we would like to discuss the most relevant ones. In our opinion, this micromachining method may have greater reliability and repeatability compared to external damper methods [21,22]. The latter uses special opto-mechanical components (prism or UV glue) to scatter modes with lower azimuthal indices on the microsphere surface, which are more complex to manufacture and apply in practice. Other selection methods based on the nonlinear properties of the microcavity medium, where mode excitation occurs due to a specific distribution of pump in active axially symmetric cavities, such as those that are disk shaped [23] or bottle shaped [24]. The latter can achieve very high suppression of side peaks (20 dB), but requires the presence of active material in the microsphere [25] for implementation. This is an interesting approach for combining it with the method presented in this work, in order to obtain microlasers with single-frequency generation.

One of the obvious disadvantages of this method is the reduction in the main parameter of the spherical resonator—Q-factor—by about two orders of magnitude. Such a large decrease may be due to several reasons. Firstly, the contamination of the surface with ablation products can occur, which leads to mode scattering. To reduce this effect, it is necessary to protect the surface of the microsphere; for example, this can be achieved with a layer of photoresist, which can later be chemically removed. Secondly, the trenches themselves create losses for all modes, including the fundamental mode, since the mode profile is infinite in the polar direction.

A special feature of the method is the strong dependence of the parameters of the resulting pattern on the position of the taper between the trenches. The positioning accuracy affects the losses for the excited modes and, apparently, this is the main reason for the relatively small ordering of the Q-factors as a function of gap size G.

## 7. Conclusions

This paper investigates a method for mode selection in a spherical microresonator by creating specialized scatterers on its surface. The scatterers were fabricated using ablation of the resonator material with femtosecond laser pulses. A phenomenological theory describing observed Fano resonances is presented. As a result of method application, a significant reduction in the number of resonances is demonstrated. For the non-inverted interference pattern, the results of [17] are confirmed, and the mode reduction coefficient in our case was found to be 180. This is because, unlike the previous work, a microsphere with a larger eccentricity (0.31) was used, and excitation was performed with a thinner taper, which led to a greater number of resonances in an unmodified microsphere. Therefore, there was no degeneracy of modes by azimuthal index in wavelength, which resulted in increased spectral density in the case of unperturbed microsphere. Additionally, for the first time in such a configuration, spectral cleaning was also achieved for the inverted Fano resonance in the form of transmission peaks, with a mode reduction coefficient of 60, including single mode regime with 3 dB side peak suppression. The obtained results can be used for creating narrowband filters, new schemes of mode selection in fiber laser resonators, and for the development of new optical sensors based on microresonators.

## Figures and Tables

**Table 1:** Parameters of microspheres with trenches.

№	G [μm]	Non-Inverted *Q* (Reduction)	Inverted *Q* (Reduction)
1	20	$5.5\;\times 10^{4}$ (140)	$2.7\;\times 10^{4}$ (26)
2	25	$3.1\;\times 10^{4}$ (162)	$4.2\;\times \;10^{4}$ (32)
3	30	$1.3\;\times \;10^{5}$ (180)	$2.4\;\times \;10^{4}$ (60)

## Data Availability

The original contributions presented in this study are included in the article. Further inquiries can be directed to the corresponding author.

## Supplementary Materials

The following supporting information can be downloaded at https://www.mdpi.com/article/10.3390/mi17030381/s1, Figure S1: Simulation results in 2D and 3D case of spherical microresonator excitation with a diameter of 40 μm by a taper of different diameters, Figure S2: The results of calculating the power overlap integrals of various modes with the trenches for different gaps.
