# Supplementary material for: Fs-Ablated Trenches on the Surface of Microsphere for Whispering Gallery Modes Cleaning
Source: Micromachines (Basel). 2026 Mar 21;17(3):381. doi: 10.3390/mi17030381 (PMC13029093; doi:10.3390/mi17030381)
Supplement: Supplementary file 1 [file micromachines-17-00381-s001.zip › micromachines-4206835-supplementary.pdf]

Supplementary materials

"Fs-ablated trenches on the surface of microsphere for whispering gallery modes cleaning"

Hiba A. Rizk, Viktor A. Simonov, Vadim S. Terentyev, Vladislav E. Fedyaj, Andrey E. Simanchuk, Alexander V. Dostovalov, Sergey A. Babin

On the issue of interference pattern formation

The formation of inverted and non-inverted spectra in taper-microsphere coupling system is demonstrated in Fig. S1 in a 2D model for fused silica microsphere with a diameter of 40 µm. Such a spectral calculation in 3D is extremely labor-intensive and requires significant computing resources. However, the results of the 2D calculation qualitatively explain the processes occurring in 3D.

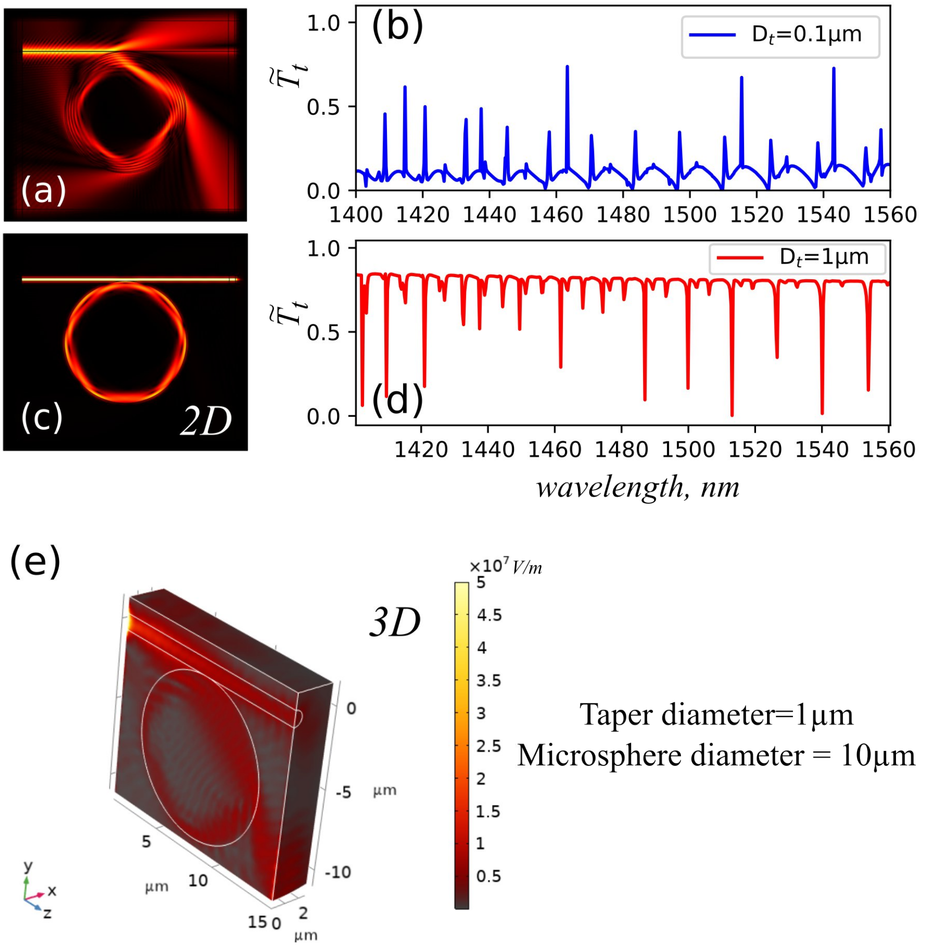

Fig. S 1. Simulation results in 2D and 3D case of spherical microresonator excitation with a diameter of 40 µm by a taper of different diameters: (a) –D_t = 0.1 µm; (b) – inverted transmission spectrum; (c) – D_t= 1 µm; (d) – non-inverted transmission spectrum and (e) - with a diameter of 10 µm and D_t= 1µm, respectively.

Fig. S1(a) shows the calculated field modulus for a very thin taper with D_t = 0.1 μm at a wavelength of 1550 nm. Fig. S1(b) shows the taper's transmission spectrum for this case. As can be seen, it exhibits high peaks and low background. The low background is due to $T_t \rightarrow 0$, and simultaneously, losses for higher radial modes increase, causing them to exit the microsphere upon refraction from the interface. This occurs because the angle γ of incidence on the surface inside the microsphere becomes smaller than the angle of total internal reflection $\gamma < \gamma_{TIR}$, which is determined by the angle and entry point of the main energy flux from the taper into the microsphere.

Notably, the entry point of light into the microsphere is offset from the point of geometric contact between the taper and the microsphere and is closer to the source. This contributes to the refraction of light at the interface between the air and the inner surface of the microsphere. The curvature of the microsphere surface reduces the angle of incidence compared to the approximate value, as the light originates at the point of contact with the taper. Such a small taper diameter is required to obtain an inverted pattern only in the 2D case.

We also performed a single-wavelength simulation of a 3D case for a unmodified microsphere with a diameter of 10 μm (Fig. S1(e)). The effect of internal refraction of radiation on the microsphere surface is shown at larger taper diameters, close to the experimental value of D_t = 1 μm.

Figure S1(c) demonstrates a thicker taper, D_t = 1 μm, at 1550 nm, and the transmission spectrum (Fig. S1(d)) becomes typical and noninverted form. This change is due to the fact that radiation from the taper mode enters the microsphere almost along the microsphere's surface and then propagates due to total internal reflection.

Trench losses

Fig. S2 shows the calculation of the energy overlap integrals for modes with different azimuthal indices for a microsphere with a diameter of 160 microns with a surface of grooves presented in the text of the article (Fig. 2(d)). Fig. S2(a) shows the projection of the microsphere mode field onto the surface of the grooves. To estimate the losses, it is assumed that the mode experiences scattering losses when it falls on the groove, the proportion of these losses should be proportional to the spatial overlap integral of the mode's energy (Fig. S2(b)).

Fig. S2(b) shows the overlap integrals. The ratio of overlap integrals is important here for modes with indices $p = l - m = 0, 2, 4$ (antisymmetric modes with $p = 1, 3$ are not excited due to the zero overlap integral with the symmetric field of the fundamental taper mode). It can be seen that

the overlap integral is 2 orders of magnitude larger for the mode relative to the main mode, i.e. larger losses are expected for modes with higher azimuthal indices ($p > 0$) than for modes with $p = 0$.

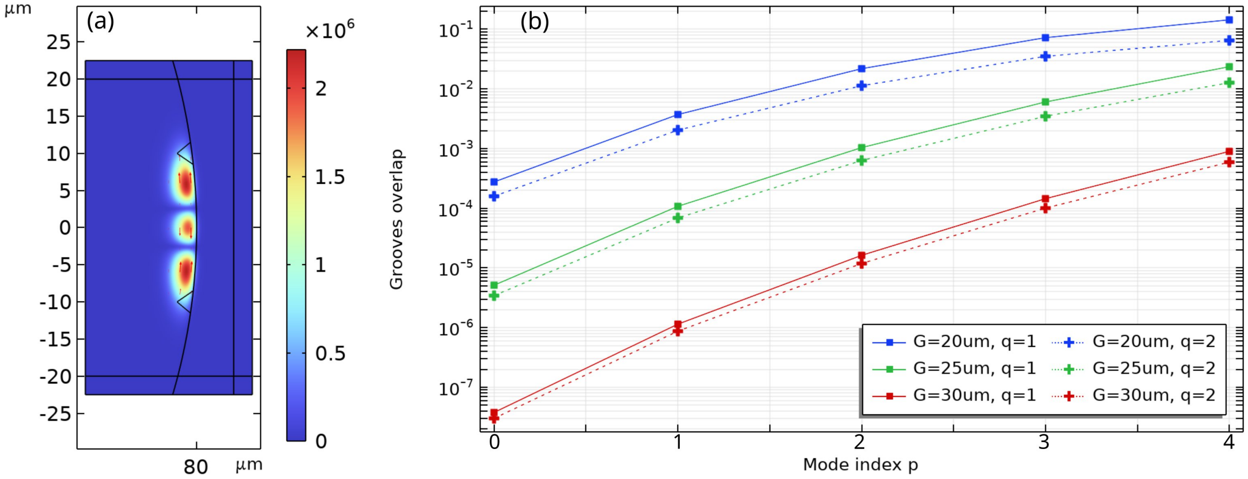

Fig. S 2. The results of calculating the power overlap integrals of various modes with the trenches for different gaps.
